# Association between albumin infusion and outcomes in patients with acute kidney injury and septic shock

**DOI:** 10.1038/s41598-021-03122-0

**Published:** 2021-12-16

**Authors:** Chenglong Ge, Qianyi Peng, Wei Chen, Wenchao Li, Lina Zhang, Yuhang Ai

**Affiliations:** 1 Department of Critical Care Medicine, Xiangya Hospital, Central South University, Changsha, 410008 People’s Republic of China; 2 National Clinical Research Center for Geriatric Disorders, Xiangya Hospital, Central South University, Changsha, 410008 People’s Republic of China

**Keywords:** Infectious diseases, Kidney diseases

## Abstract

Septic shock with acute kidney injury (AKI) is common in critically ill patients. Our aim was to evaluate the association between albumin infusion and outcomes in patients with septic shock and AKI. Medical Information Mart for Intensive Care (MIMIC)-III was used to identify patients with septic shock and AKI. Propensity score matching (PSM) was employed to balance the baseline differences. Cox proportional hazards model, Wilcoxon rank-sum test, and logistic regression were utilized to determine the associations of albumin infusion with mortality, length of stay, and recovery of kidney function, respectively. A total of 2861 septic shock patients with AKI were studied, including 891 with albumin infusion, and 1970 without albumin infusion. After PSM, 749 pairs of patients were matched. Albumin infusion was associated with improved 28-day survival (HR 0.72; 95% CI 0.59–0.86; *P* = 0.002), but it was not difference in 90-day mortality between groups (HR 0.94; 95% CI 0.79–1.12; *P* = 0.474). Albumin infusion was not associated with the renal function recovery (HR 0.91; 95% CI 0.73–1.13; *P* = 0.393) in either population. Nevertheless, subgroup analysis showed that albumin infusion was distinctly associated with reduced 28-day mortality in patients with age > 60 years. The results need to be validated in more randomized controlled trials.

## Introduction

Septic shock syndrome resulting from systemic inflammation and excessive host immune responses to infection is a top cause of death in hospitalized patients, with 40–50% mortality^1,2^. Acute kidney injury (AKI) is a common complication of septic shock^3^. It accounts for approximately 80% of all septic shock inpatients^4^. Early reasonable fluid resuscitation followed by vasopressor is one of the most important strategies for initial treatment in patients with septic shock and AKI.

Human serum albumin (HSA) has been considered for the treatment of septic shock in initial fluid resuscitation because of its advantages of restoring effective volume and maintaining colloidal osmotic pressure^5,6^. Whether albumin can improve patient-centered outcomes beyond expanding blood volume, however, is uncertain. Based on weak recommendation, the latest Surviving Sepsis Campaign guidelines suggested albumin for the initial resuscitation when patients require substantial amounts of crystalloids^7^. At present, the use of albumin for fluid resuscitation in the treatment of septic shock remains controversial. A multicenter randomized controlled trial (RCT) showed that albumin replacement in addition to crystalloids, as compared with crystalloids alone, did not improve the rate of survival at 28 and 90 days^8^. But a subsequent meta-analysis of RCTs found that albumin infusion was associated with reduced 90-day mortality^9^. Nevertheless, there is little available evidence from RCTs or guidelines to support its practice in AKI among patients with septic shock. Therefore, further studies are needed to investigate the efficacy of albumin infusion in patients with septic shock and AKI.

Thus, this study mainly discussed the relationship between albumin infusion and outcomes in patients with septic shock and AKI. Furthermore, propensity score matching (PSM) would be used to reduce potential imbalance of baseline characteristic between the albumin and non-albumin groups. The conclusions drawn from such a design will be more reliable.

## Results

### Basic characteristics

**Figure 1:**
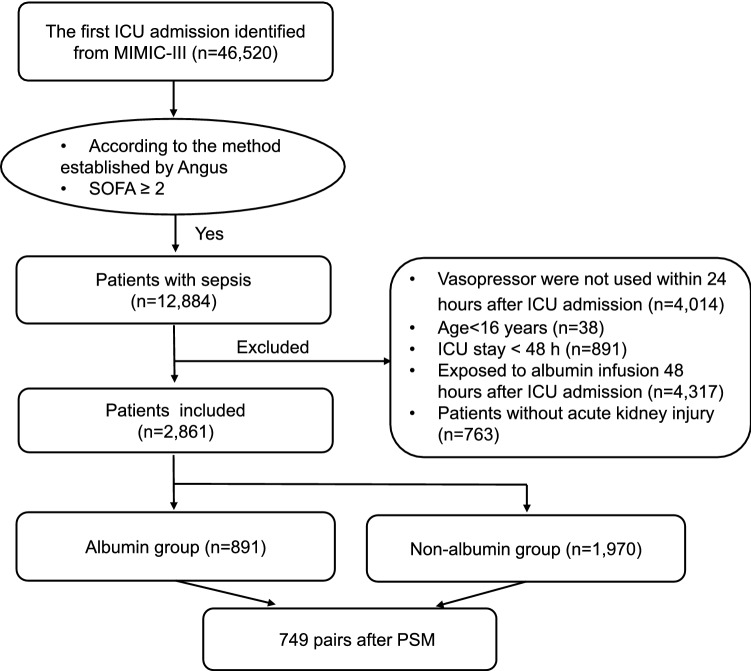
Flowchart of patient inclusion. *MIMIC-III* Multiparameter Intelligent Monitoring in Intensive Care Database III; *SOFA* sequential organ failure assessment; *ICU* intensive care unit; *PSM* propensity-score matching.

A total of 46,520 patients with the first ICU admission were extracted from the MIMIC-III database. A total of 12,884 patients fitted the definition of sepsis-3.0 within 48 h after ICU admission. A total of 2861 patients were examined in the analysis based on the exclusion criteria. Of the study cohort, 891 patients were administrated with albumin infusion in the first 48 h in the ICU, while the remaining 1,970 patients did not receive albumin infusion (Fig. 1).

The baseline characteristics of the two groups before PSM are presented in Table 1. The mean age was slightly lower (69 vs. 71), and the SOFA score and SAPSII score was higher in the albumin group at admission, in comparison with the non-albumin group. There were also significant differences in AKI stages between the two groups. Patients with hypertension, coagulopathy, liver cirrhosis, or obesity were more likely to be given the albumin group. The levels of PT, and PH were lower, while the levels of platelet, glucose, hemoglobin, and lactate were statistically higher in the non-albumin set compared to the albumin set. The uses of RRT, inotropes, and mechanical ventilation were much more common in the albumin group. The volume of daily crystalloid infusion was more, and the urine output was less in the albumin group.

**Table 1:** Baseline characteristics between groups before propensity score matching.

Variables	Overall	Non-albumin group	Albumin group	*P* value	SMD
	2861	1970	891		
Gender, male (%)	1544 (54.0)	1089 (55.3)	455 (51.1)	0.04	0.085
Age (median [IQR])	70 [59, 80]	71 [60, 80]	69 [57, 78]	< 0.001	0.161
Weight (median [IQR])	80 [68, 97]	80 [68, 97]	81 [68, 97]	0.674	0.008
**Ethnicity (%)**				0.084	0.107
Asian	60 (2.1)	47 (2.4)	13 (1.5)		
Black	165 (5.8)	123 (6.2)	42 (4.7)		
White	2056 (71.9)	1393 (70.7)	663 (74.4)		
Other	580 (20.3)	407 (20.7)	173 (19.4)		
SOFA (median [IQR])^b^	8 [5, 10]	7 [5, 10]	8 [6, 11]	< 0.001	0.321
GCS (median [IQR])^b^	9 [5, 14]	9 [6, 14]	8 [3, 12]	< 0.001	0.238
SAPSII (median [IQR])^b^	48 [39, 58]	47 [39, 57]	49 [40, 60]	0.001	0.142
RRT (%)	214 (7.5)	131 (6.6)	83 (9.3)	< 0.015	0.098
Ventilation (%)	2410 (84.2)	1594 (80.9)	816 (91.6)	< 0.001	0.314
Inotropes use (%)	271 (9.5)	147 (7.5)	124 (13.9)	< 0.001	0.21
Other colloid input, n (%)	66 (2.3)	41 (2.1)	25 (2.8)	0.289	0.047
**Co-morbidities (%)**					
*AKI stage (%)*				< 0.001	0.291
1	496 (17.3)	383 (19.4)	113 (12.7)		
2	1376 (48.1)	986 (50.1)	390 (43.8)		
3	989 (34.6)	601 (30.5)	388 (43.5)		
CKD	417 (14.6)	285 (14.5)	132 (14.8)	0.852	0.01
Congestive heart failure	1203 (42.0)	912 (46.3)	291 (32.7)	< 0.001	0.282
End stage renal disease	194 (6.8)	137 (7.0)	57 (6.4)	0.639	0.022
Liver cirrhosis	113 (3.9)	30 (1.5)	83 (9.3)	< 0.001	0.349
Cardiovascular diseases	2051 (71.7)	1450 (73.6)	601 (67.5)	0.001	0.135
Hypertension	1487 (52.0)	969 (49.2)	518 (58.1)	< 0.001	0.18
Chronic pulmonary diseases	688 (24.0)	478 (24.3)	210 (23.6)	0.722	0.016
Diabetes	228 (8.0)	157 (8.0)	71 (8.0)	1	< 0.001
ARDS	19 (0.7)	15 (0.8)	4 (0.4)	0.481	0.04
Coagulopathy	661 (23.1)	354 (18.0)	307 (34.5)	< 0.001	0.382
Obesity	224 (7.8)	138 (7.0)	86 (9.7)	0.018	0.096
Anemia	141 (4.9)	88 (4.5)	53 (5.9)	0.109	0.067
Mean heartrate (median [IQR])^b^	88 [78, 101]	88 [78, 101]	88 [79, 101]	0.435	0.053
Mean MAP (median [IQR])^b^	72 [67, 77]	72 [67, 77]	71 [66, 77]	0.02	0.098
Platelet (median [IQR])^a^	190 [128, 274]	199 [138, 283]	158 [109, 247]	< 0.001	0.232
Creatinine (median [IQR])^a^	1.2 [0.8, 2.1]	1.2 [0.8, 2.1]	1.2 [0.8, 2.1]	0.996	0.078
Glucose (median [IQR])^a^	125 [103, 159]	127 [104, 166]	120 [102, 148]	< 0.001	0.234
Hemoglobin (median [IQR])^a^	10.2 [9.2, 11.6]	10.4 [9.2, 11.8]	9.9 [8.9, 11.1]	< 0.001	0.258
PT (median [IQR])^a^	14.7 [13.4, 17.0]	14.5 [13.2, 16.5]	15.3 [13.7, 18.5]	< 0.001	0.098
WBC (median [IQR])^a^	12 [8.5, 17]	12 [8.6, 17.2]	11.9 [8.3, 16.8]	0.242	0.043
Lactate (median [IQR])^a^	1.9 [1.3, 3.0]	2.0 [1.4, 3.0]	1.7 [1.2, 2.8]	< 0.001	0.083
PH (median [IQR])^a^	7.3 [7.3, 7.4]	7.3 [7.3, 7.4]	7.4 [7.3, 7.4]	0.007	0.082
Crystalloid input (median [IQR])^b^	1500 [0, 3300]	1000 [0, 3000]	2300 [500, 4500]	< 0.001	0.378
Urine output (median [IQR]) ^b^	566 [294, 859]	592 [297, 915]	492 [287, 719]	< 0.001	0.258

*SOFA* Sequential Organ Failure Assessment, *SAPSII* Simplified Acute Physiology Score II, *GCS* Glasgow Coma Scale, *RRT* renal replacement therapy, *AKI* acute kidney injury, *CKD* chronic kidney disease, *ARDS* acute respiratory distress syndrome, *MAP* mean arterial pressure, *PT* prothrombin time, *WBC* white blood cell, *IQR* interquartile range, *SMD* standardized mean difference.

^a^The initial value during the first 24 h after ICU admission.

^b^The values were calculated during the first 24 h after ICU admission.

### Relationship between albumin infusion and outcomes

**Figure 2:**
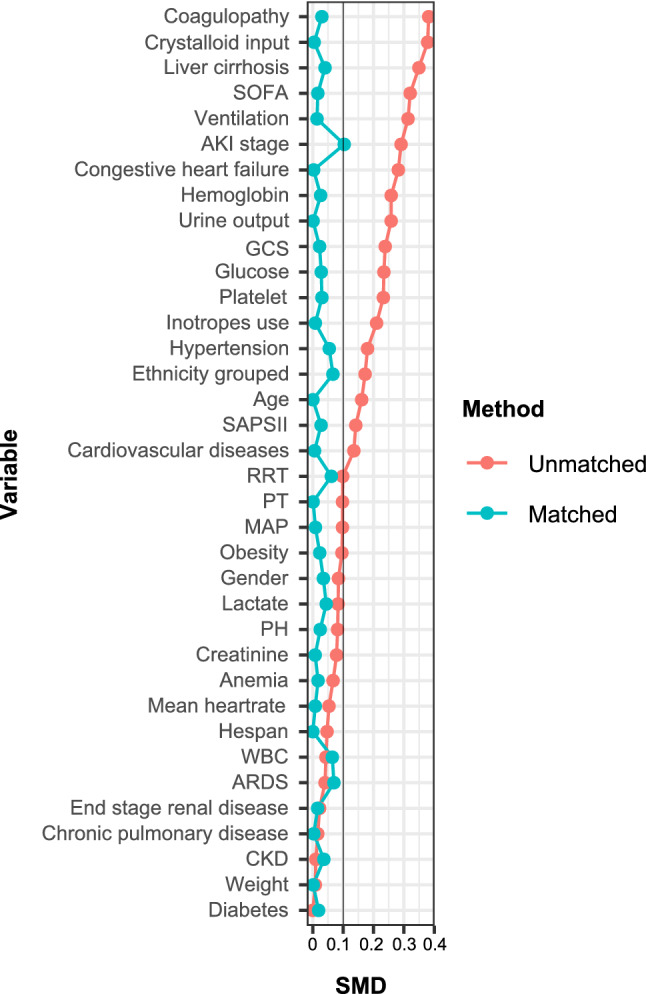
Standardized mean difference (SMD) of variables before and after propensity score matching. *SOFA* Sequential Organ Failure Assessment; *AKI* acute kidney injury; *GCS *Glasgow Coma Scale; *SAPSII* Simplified Acute Physiology Score II; *RRT* renal replacement therapy; *PT* prothrombin time; *MAP* mean arterial pressure; *WBC* white blood cell; *ARDS* acute respiratory distress syndrome. Statistical analysis was performed using R 4.0.0 software.

After PSM, 749 patients who did not receive albumin infusion were matched with 749 patients who received albumin infusion. The imbalance between the two groups was significantly reduced after PSM, and SMDs of all variables were less than 10% (Table S3; Fig. 2).

In the pre-matched cohort, albumin infusion was related to improved mortality at 28 days (HR 0.75; 95% CI 0.63–0.90; *P* = 0.002), but there was no difference in 90-day mortality between groups (HR 0.92; 95% CI 0.79–1.06; *P* = 0.24), following adjustment of the confounders with *P* < 0.05 in univariate analysis (Table 2; Supplementary Table S2). The impact of albumin infusion on the renal function recovery was assessed by the logistic regression model, and the result showed that albumin infusion was associated with delayed recovery of renal function (HR 0.78; 95% CI 0.66–0.92; *P* = 0.004). In addition, we found that albumin infusion was associated with extended LOS in ICU and hospital (Table 2).

**Table 2:** Association between albumin infusion and clinical outcomes in patients with septic shock and acute kidney injury.

Outcomes	Non-albumin	Albumin	*P*-value	HR (95%CI)
**Pre-matched cohort**	**n = 1970**	**n = 891**		
Primary outcome				
28-day mortality, n (%)^a^	533 (27.1)	231 (25.9)	0.002	0.75 (0.63–0.90)
Secondary outcomes				
90-day mortality, n (%)^a^	684 (34.7)	333 (37.4)	0.24	0.92 (0.79–1.06)
Recovery of renal function, n (%)^b^	1291 (65.5)	514 (57.7)	0.004	0.78 (0.66–0.92)
Length of hospital stay (days, median [IQR])^c^	12.8 [8, 20]	18.3 [11, 29]	< 0.001	
Length of ICU stay (days, median [IQR])^c^	5.9 [3.4, 11.2]	8.8 [4.2, 16.8]	< 0.001	
**Post-matched cohort**	**n = 749**	**n = 749**		
Primary outcome				
28-day mortality, n (%)^a^	224 (29.9)	184 (24.6)	0.002	0.72 (0.59–0.86)
Secondary outcomes				
90-day mortality, n (%)^a^	268 (35.8)	275 (36.7)	0.474	0.94 (0.79–1.12)
Recovery of renal function, n (%)^b^	487 (65.0)	446 (59.5)	0.345	0.82 (0.69–1.11)
Length of hospital stay (days, median [IQR])^c^	13.45 [8.1, 20.2]	18 [10.8, 28.4]	< 0.001	
Length of ICU stay (days, median [IQR])^c^	6.4 [3.7, 12.8]	8.2 [4.2, 16.3]	0.001	

*IQR* interquartile range, *ICU* intensive care unit, *HR* hazard ratio, *CI* confidence interval.

^a^Cox proportional hazard models were used to assess the impact of albumin infusion on mortality outcomes adjusting for confounders selected from *P-*value < 0.05 in univariate analysis.

^b^Recovery of renal function was defined as being discharged from ICU with serum creatinine below 1.5 times the baseline value and normal urine output (> 0.5 ml/kg/h). Impact of albumin infusion on the recovery of renal function was assessed using the logistic regression model adjusting for age, SAPSII score, and RRT use.

^c^Wilcoxon rank sum test was used to assess the association between albumin infusion and length of stay.

In the post-matched cohort, similarly to the results before PSM, albumin infusion was associated with improved 28-day survival (HR 0.72; 95% CI 0.59–0.86; *P* = 0.002), and there was also no difference in 90-day mortality between groups (HR 0.94; 95% CI 0.79–1.12; *P* = 0.474) (Table 2). Nevertheless, the recovery of renal function was not statistically different between the albumin group and the non-albumin group (HR 0.82; 95% CI 0.69–1.11; *P* = 0.345) (Table 2). The albumin infusion was also associated with longer LOS in ICU and hospital (Table 2).

Taking the dose of albumin into consideration, we found that receiving 20–50 g in the first 48 h after ICU admission was associated with a reduced risk of 28-day mortality when compared with the non-albumin group (HR 0.59; 95% CI 0.39–0.88; *P* = 0.009) (Table S4). High-dose (> 50 g/48 h) or low-does (≤ 25 g/48 h) albumin were not associated with improved mortality of patients with septic shock and AKI (Table S4). Furthermore, in order to explore the effects of other colloid solutions other than albumin on 28-day mortality, cox regression models were performed. The result showed that use of albumin was associated with decreased 28-day mortality, but no apparent survival advantage was observed in the hydroxyethyl starch (HES) group (Table S5).

### Subgroup analysis

**Figure 3:**
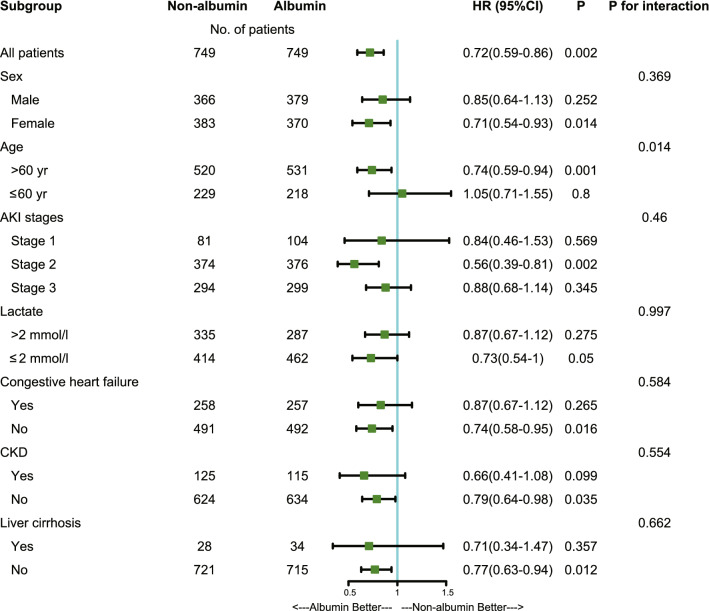
The association between albumin infusion and 28-day mortality in subgroups. *AKI* acute kidney injury; *HR* hazard ratio; *CI* confidence interval; *CKD* chronic kidney disease. Statistical analysis was performed using R 4.0.0 and Stata 15.1 software.

The relationship between albumin infusion and 28-day mortality in subgroups is shown in Fig. 3. The effect of albumin was consistent across all subgroups. In addition, we found an interaction between age and albumin infusion (*P* for interaction = 0.014, without adjustment for multiple comparisons) after PSM. The results indicated that there was a clear correlation between albumin infusion and improved 28-day survival among those patients with age > 60 years.

## Discussion

To our knowledge, this is one of the first studies comparing the association of albumin infusion and outcomes in patients with septic shock and acute kidney injury. Our results suggested that albumin infusion was associated with improved 28-day mortality for patients with AKI and septic shock, but it did not significantly affect the 90-day mortality. The results of our study also suggested that no significant correlation was found between albumin infusion and the recovery of renal function after PSM. Subgroup analysis showed that albumin infusion in comparison to other crystalloid fluid might be better in older population for those patients with AKI and septic shock.

Although albumin has been widely used for patients with septic shock, the evidence to support its effectiveness has not been fully established, especially in septic shock patients with AKI. A multi-center randomized controlled trial (ALBIOS study) found that albumin administration in addition to crystalloids, as compared with crystalloids alone, did not improve 28-day and 90-day mortality rates in sepsis^8^. Regarding the association between albumin and septic shock, a meta-analysis included five studies showed 90-day mortality of patients with septic shock decreased significantly^9^. These results were partially consistent with our findings. Unfortunately, the status of AKI was not included in these studies. The 2016 surviving sepsis guideline suggested the use of albumin for patients with sepsis and septic shock when patients require substantial amounts of crystalloids (weak recommendation, low quality of evidence)^7^, but there is a lack of evidence for septic shock patients with AKI. In this regard, our study provides timely evidence that albumin infusion is potentially beneficial for patients with septic shock and AKI (Fig. 3). This may be achieved by stabilizing endothelial cell function and reducing sympathetic excitation, endotoxemia and oxidative stress^10^. A previous study^11^ indicated that albumin infusion can decrease plasma norepinephrine levels and improve renal function in patients with cirrhosis-induced AKI. Consistent with our study, a previous randomized controlled trial (SAFE Study) found that administration of albumin compared to saline did not impair renal function and may have decreased the risk of death among patients with severe sepsis^12^.

The finding that albumin infusion was associated with improved 28-day mortality but not with 90-day mortality can be explained as follows: First, SOFA score and SAPSII use previous physiological parameters and health status from the first 24-h of ICU admission. Compared with 28-day mortality, 90-day mortality may be more influenced by chronic health status and complication than by acute physiological parameters. Second, it is generally speculated that the longer the ICU stay, the more complication occurs, resulting in a poor therapeutic effect. Therefore, albumin infusion failed to show a 90-day mortality benefit. Besides, why the better survival 28-day was not observed in young adults? Only in elderly? One of the possible explanations is that older people have less vascular response and vascular compliance, resulting in greater need for albumin to maintain vascular tone than younger people. However, these results should be considered exploratory and require further exploration before reaching definitive conclusions.

Theoretically, human albumin is the most plentiful plasma protein. Because of its macromolecular structure, HSA plays a key role in the regulation of microvascular hydrodynamics^13^. In addition, HSA is a multifunctional plasma protein, with antioxidant and anti-inflammatory properties^10^, as well as buffering capacity to regulate acid-based balance^14^. Experimental results of Geoffroy et al.^5^ showed that albumin infusion can improve endothelial function in patients with septic shock. At present, international guidelines do not recommend albumin as the first choice for resuscitation in severe sepsis or septic shock^7,15^. However, until further studies reach the opposite conclusion, we believe that it is reasonable to consider albumin as a potential resuscitation fluid. At the same time, in clinical practice, most patients with septic shock will continue to receive mixed liquid resuscitation rather than single liquid resuscitation.

Taking albumin dose into account, we found that 20–50 g of albumin administered within 48 h after ICU hospitalization was associated with a reduced risk of 28-day mortality, but no apparent survival advantages were observed between high-dose (> 50 g/48 h) and low-does (≤ 25 g/48 h). Besides, our study showed HES did not improve 28-day survival of patients with septic shock and AKI, which were consistent with those previously reported^16,17^.

However, there are still several limitations to our study. Firstly, the current research is based on a clinical electronic medical record that contains some missing values. To improve statistical power, multiple imputations were used to decrease the risk of deviation due to missing values. Secondly, we did not report the range of albumin concentrations in this study. The effect of the concentrations of albumin on mortality is unclear. Thirdly, we performed multiple subgroup analyses, which may yield false positive results. However, P for interaction showed the same result after PSM, which increased the robustness of the findings. Fourth, due to the characteristics of a retrospective research, the relationship between the albumin infusion and mortality can only be interpreted indirectly, which may only provide preliminary evidence for further investigation. In addition, as vasoactive-inotropic score (VIS) is hard to be extracted from database, we did not compare the hemodynamic status between two group. However, we have included use of vasopressors or inotropic agents in the baseline characteristics and PSM. Besides, the effect of different crystalloid like Lactated Ringer's solution vs. normal saline was not discussed and analyzed for various reasons. Finally, apart from concerns regarding effects on renal function, the adverse events of albumin infusion were not reported in our study. While it is difficult to extract information on adverse events from MIMIC database, this needs to be further explored in prospective trials.

In conclusion, albumin infusion was significantly associated with improved 28-day mortality among patients with septic shock and AKI, but it was not associated with improved 90-day survival. Albumin infusion in comparison to other crystalloid fluid might be better in older population. The results need to be validated in more randomized controlled trials.

## Material and methods

### Data source

The data utilized in this retrospective study was from MIMIC-III, which is an openly accessible US-based critical care repository^18^. The MIMIC III database includes clinical information on patients hospitalized from 2001 to 2012 in the adult ICUs of Beth Israel Deaconess Medical Center. It is also approved by the Massachusetts Institute of Technology Institutional Review Boards. Patients were selected using the PostgreSQL 9.6 software from the latest version (MIMIC-III v1.4), which was released on the 2nd of September 2016.

### Participants and definition

PgAdmin (version 4.1, Bedford, USA) was used to mine data from the MIMIC III data bank. The study inclusion criteria included patients (1) that were diagnosed with sepsis; (2) patients with septic shock; (3) patients with AKI. According to the Sepsis-3 definition, sepsis was defined as patients with suspected or verified infection, plus an acute change in total *SOFA* scores ≥ 2^19^. Sepsis was diagnosed according to the method established by Angus^20^, identifying patients based on the ICD-9 code in the MIMIC-III database. All sepsis patients who were supported with vasopressor within 24 h after ICU admission were defined as patients with septic shock^21^. AKI was defined according to the Kidney Disease: Improving Global Outcomes (KDIGO) criteria^22^. KDIGO criteria are defined as follows: the elevated level in serum creatinine concentration is 1.5 times greater than baseline, or the rise in serum creatinine concentration is higher than 0.3 mg/dL (≥ 26.5 µmol/L) within 48 h, or the volume of urine is less than < 0.5 mL/kg/h for 6 h. AKI stages were determined by both serum creatinine and urine output during the first 48 h of ICU according to the KDIGO criteria.

Patients with age < 16 years old, and those who were discharged or died within 48 h after ICU admission were excluded. Patients were also excluded from the study if they received albumin infusion 48 h after ICU admission. For a patient with more than one ICU admissions, only the first admission was included. According to the albumin infusion status within 48 h after ICU admission, the participants were separated into two groups: albumin group (intervention) and non-albumin group (control).

### Demographic and laboratory variables

The following data were extracted from MIMIC-III for the first 24 h of ICU using Structured Query Language (SQL): gender, age, weight, mean arterial pressure (MAP), baseline laboratory data (the first measurement on the first day), sequential organ failure assessment (SOFA) score, simplified acute physiology score II (SAPS II), Glasgow coma score (GCS), comorbidities, use of inotropes, vasopressor, crystalloid, colloid, renal replacement therapy (RRT), and mechanical ventilation. SOFA, SAPSII and GCS were calculated as described in previous studies^23,24^. The total does of crystalloid during the first 24 h of ICU admission was included in this analysis. RRT modalities included continuous RRT (CRRT), daily conventional dialysis (daily-IHD), and prolonged intermittent RRT (PIRRT). Definition of obesity is BMI greater than 30^25^. Coagulopathy was defined as prothrombin time prolonged by 3 s or activated partially thrombin time prolonged by 5 s^26^. Cardiovascular diseases included arrhythmias, angina pectoris, myocardial infarction, and heart failure. Chronic lung disease (CLD) mainly included chronic obstructive pulmonary disease (COPD) and interstitial lung disease.

No more than 15% missing values were recorded in all variables (Supplementary Table S1). Missing values were filled by single imputation or linear regression as appropriate (see additional file 1: Table S1).

### Endpoints

The primary end point was 28-day mortality. The secondary end points are 90-day mortality, recovery of renal function, length of stay (LOS) in hospital, and LOS in ICU. Recovery of renal function was defined if the urine output on discharge is normal (> 0.5 ml/kg/h for 24 h) and a return to a creatinine level of 150% as of the baseline on ICU discharge^24^.

### Statistics analysis

Continuous variables are expressed as median [interquartile range (IQR)] due to their non-normal distribution. The differences between groups were determined by the Mann–Whitney *U* test. Categorical variables are shown as frequencies and percentages. The comparisons were performed by the χ^2^ test or Fisher's exact test as appropriate.

To balance the baseline differences, propensity-score matching (PSM) was conducted with a caliper width of 0.2 logits of the standard difference. Patients were divided using 1:1 nearest neighbor matching, so that each person in the albumin group was matched with those in the non-albumin group. The standardized mean difference (SMD) was used to assess the effectiveness of PSM^27^ (Fig. 2).

The Cox regression model was performed to assess the relationship between albumin infusion and mortality after adjustment for confounding variables with *P* < 0.05 in univariate analysis (Supplementary Table S2. The logistic regression model was used to assess the impact of albumin infusion on the recovery of renal function after adjusting for age, SAPSII score, and RRT use. LOS in hospital and LOS in ICU were compared by Wilcoxon rank-sum test between two groups.

Various subgroups were classified by different age, lactate, AKI stage, congestive heart failure, chronic kidney disease (CKD) and liver cirrhosis. The association between the daily dose of albumin and 28-day mortality was also assessed (Supplementary Table S4). Table S5 showed that the correlation between other colloid solutions and 28-day mortality. Multivariate analysis by Cox regression was used in subgroup analyses after adjusting for potential confounders, which were performed after PSM.

Statistical analysis was carried out using software Stata 15.1 (https://www.stata.com/) and R 4.0.0 (https://www.r-project.org/) in the Windows operative system. Statistical significance was determined when the *p* value is less than 0.05.

### Ethics approval and consent to participate

The study was an analysis of a third-party anonymized publicly available database with pre-existing institutional review board (IRB) approval. The Institutional review boards at the Beth Israel Deaconess Medical Center (protocol 2001-P-001699/14) and Massachusetts Institute of Technology (protocol 0403000206) have approved the data collection and the use of MIMIC-III for research purposes and granted waiver of informed consent. All methods were carried out in accordance with relevant guidelines and regulations.

## Supplementary Information

Supplementary Table S1.

Supplementary Table S2.

Supplementary Table S3.

Supplementary Table S4.

Supplementary Table S5.

## Data Availability

All data and material were available at https://mimic.mit.edu/.
