# Supplementary material for: Association between albumin infusion and outcomes in patients with acute kidney injury and septic shock
Source: Sci Rep. 2021 Dec 16;11:24083. doi: 10.1038/s41598-021-03122-0 (PMC8677767; doi:10.1038/s41598-021-03122-0)
Supplement: Supplementary file 1 — Supplementary Table S1. [file 41598_2021_3122_MOESM1_ESM.docx]

**Table S1. All screening variables contained less than 15% missing values**

| **Variables** | **Missing number (%)** |
| --- | --- |
| Gender | 0 (0) |
| Age | 0 (0) |
| Weight | 6 (0.1) |
| ethnicity | 0 (0) |
| SOFA ^b^ | 0 (0) |
| SAPSII ^b^ | 0 (0) |
| GCS ^b^ | 98 (2.2) |
| RRT | 0 (0) |
| Ventilation | 0 (0) |
| Inotropes use | 0 (0) |
| Other colloid use | 0 (0) |
| AKI | 0 (0) |
| AKI stage | 3 (0.07) |
| CKD | 0 (0) |
| Congestive heart failure | 0 (0) |
| End stage renal failure | 0 (0) |
| Liver cirrhosis | 0 (0) |
| Cardiovascular disease | 0 (0) |
| Hypertension | 0 (0) |
| Chronic lung disease | 0 (0) |
| Diabetes | 0 (0) |
| ARDS | 0 (0) |
| Coagulopathy | 0 (0) |
| Obesity | 0 (0) |
| Anemia | 0 (0) |
| Mean heart rate ^b^ | 3 (0.07) |
| Mean MAP ^b^ | 0 (0) |
| Platelet ^a^ | 82 (1.8) |
| Creatinine ^a^ | 13 (0.3) |
| Urine output ^b^ | 85 (1.9) |
| Glucose ^a^ | 41 (0.9) |
| Hemoglobin ^a^ | 11 (0.2) |
| PT ^a^ | 113 (2.5) |
| WBC ^a^ | 10 (0.2) |
| PH ^a^ | 554 (12.2) |
| Lactate ^a^ | 522 (11.5) |
| Albumin use | 0 (0) |
| Albumin does | 0 (0) |
| Dextran use | 0 (0) |
| Crystalloid | 0 (0) |
| Length of hospital stay | 0 (0) |
| Length of ICU stay | 0 (0) |

**Abbreviations**: SOFA: sequential organ failure assessment, SAPSII: simplified acute physiology score II, GCS: Glasgow coma score, MAP: mean arterial pressure, CKD chronic kidney disease, ARDS: acute respiratory distress syndrome, AKI: acute kidney injury, RRT: renal replacement therapy, PT prothrombin time, WBC white blood cell

^a^ The initial values during the first 24h after ICU admission.

^b^ The values were calculated during the first 24h after ICU admission.
