# Supplementary material for: Association between albumin infusion and outcomes in patients with acute kidney injury and septic shock
Source: Sci Rep. 2021 Dec 16;11:24083. doi: 10.1038/s41598-021-03122-0 (PMC8677767; doi:10.1038/s41598-021-03122-0)
Supplement: Supplementary file 2 — Supplementary Table S2. [file 41598_2021_3122_MOESM2_ESM.docx]

**Table S2. Potential risk factors associated with 28-day mortality by univariate Cox regression analysis**

| **Variables** | **HR (95% CI)** | **P value** |
| --- | --- | --- |
| Male | 0.94 (0.85-1.1) | 0.32 |
| Age (per 20 y increase) | 1.32 (1.19-1.47) | <0.001 |
| Weight (per 10 kg increase) | 0.92 (0.89-0.95) | <0.001 |
| Ethnicity | 0.9 (0.8-1.03) | 0.122 |
| SOFA (per 1 score increase) ^b^ | 1.14 (1.12-1.16) | <0.001 |
| GCS (per 1 score increase) ^b^ | 0.98 (0.97-0.99) | 0.02 |
| SAPSII (per 1 score increase) ^b^ | 1.04 (1.03-1.04) | <0.001 |
| RRT | 1.43 (1.13-1.81) | 0.003 |
| Ventilation | 1.04 (0.85-1.26) | 0.706 |
| Inotropes use | 0.63 (0.48-0.84) | 0.002 |
| Other colloid use | 0.20 (0.07-0.53) | 0.001 |
| AKI stage (per 1 stage increase) | 1.77 (1.58-1.97) | <0.001 |
| CKD | 1.05 (0.86-1.27) | 0.651 |
| Congestive heart failure | 1.06 (0.92-1.22) | 0.454 |
| End stage renal disease | 1.13 (0.86-1.47) | 0.383 |
| Liver cirrhosis | 2.23 (1.69-2.95) | <0.001 |
| Cardiovascular diseases | 0.84 (0.72-0.98) | 0.03 |
| Hypertension | 0.74 (0.64-0.85) | <0.001 |
| Chronic lung disease | 0.95 (0.80-1.12) | 0.5 |
| Diabetes | 1.05 (0.82-1.36) | 0.698 |
| ARDS | 1.48 (0.70-3.12) | 0.3 |
| Coagulopathy | 1.64 (1.40-1.91) | <0.001 |
| Obesity | 0.52 (0.37-0.74) | <0.001 |
| Anemia | 0.79 (0.56-1.13) | 0.2 |
| Mean heartrate (per 20 times increase) ^b^ | 1.2 (1.11-1.31) | <0.001 |
| Mean MAP (per 10 mmHg increase) ^b^ | 0.71 (0.64-0.78) | <0.001 |
| Platelet (per 10 k/ul increase) ^a^ | 1 (0.99-1.1) | 0.898 |
| Bilirubin (per 1mg/dL increase) ^a^ | 1.05 (1.04-1.06) | <0.001 |
| Creatinine (per 1mg/dL increase) ^a^ | 1.09 (1.05-1.13) | <0.001 |
| Glucose (per 20 mg/dL increase) ^a^ | 1.04 (1.02-1.05) | <0.001 |
| Hemoglobin (per 5 mg/dL increase) ^a^ | 1.06 (0.88-1.28) | 0.543 |
| PT (per 5 s increase) ^a^ | 1.09 (1.07-1.11) | <0.001 |
| WBC (per 5 ×10^9/L increase) ^a^ | 1.08 (1.04-1.13) | <0.001 |
| Lactate (per 1 mmol/L increase) ^a^ | 1.15 (1.13-1.17) | <0.001 |
| PH (per 1 unit increase) ^a^ | 0.24 (0.13-0.43) | <0.001 |
| Crystalloid does (per 500 ml increase) ^b^ | 0.99 (0.98-1.01) | 0.341 |
| Urine output (per 100 ml increase) ^b^ | 0.99 (0.98-1) | 0.09 |

**Abbreviations**: SOFA: sequential organ failure assessment, SAPSII: simplified acute physiology score II, GCS: Glasgow coma score, MAP: mean arterial pressure, ARDS: acute respiratory distress syndrome, AKI: acute kidney injury, CKD: chronic kidney disease, RRT: renal replacement therapy, PT prothrombin time, WBC white blood cell.

^a^ The initial values during the first 24h after ICU admission.

^b^ The values were calculated during the first 24h after ICU admission.
