# Supplementary material for: Association between albumin infusion and outcomes in patients with acute kidney injury and septic shock
Source: Sci Rep. 2021 Dec 16;11:24083. doi: 10.1038/s41598-021-03122-0 (PMC8677767; doi:10.1038/s41598-021-03122-0)
Supplement: Supplementary file 3 — Supplementary Table S3. [file 41598_2021_3122_MOESM3_ESM.docx]

**Table S3. Comparisons between groups after propensity score matching**

| **Variables** | **Non-albumin** | **Albumin** | **P value** | **SMD** |
| --- | --- | --- | --- | --- |
|  | **749** | **749** |  |  |
| Gender, male, n (%) | 366 (48.9) | 379 (50.6) | 0.535 | 0.035 |
| Age (median [IQR]) | 69 [57,80] | 70 [58,79] | 0.892 | <0.001 |
| Weight (median [IQR]) | 80 [68,96] | 81 [67,97] | 0.882 | 0.001 |
| Ethnicity, n (%) |  |  | 0.896 | 0.066 |
| Asian | 6 (0.8) | 10 (1.3) |  |  |
| Black | 41 (5.5) | 35 (4.7) |  |  |
| Hispanic | 24 (3.2) | 23 (3.1) |  |  |
| Other | 17 (2.3) | 15 (2.0) |  |  |
| Unknown | 103 (13.8) | 104 (13.9) |  |  |
| White | 558 (74.5) | 562 (75) |  |  |
| SOFA (median [IQR]) ^b^ | 8 [6,11] | 8 [6,11] | 0.444 | 0.017 |
| GCS (median [IQR]) ^b^ | 8 [4,11] | 9 [3,13] | 0.666 | 0.002 |
| SAPAII (median [IQR]) ^b^ | 50 [40,60] | 49 [40,59] | 0.404 | 0.017 |
| RRT, n (%) | 50 (6.7) | 62 (8.3) | 0.28 | 0.061 |
| Ventilation, n (%) | 682 (91.9) | 679 (90.7) | 0.858 | 0.014 |
| Inotropes use, n (%) | 85 (11.3) | 87 (11.6) | 0.935 | 0.008 |
| Hespan use, n (%) | 24 (3.2) | 24 (3.2) | 1 | <0.001 |
| AKI stage, n (%) |  |  | 0.136 | 0.103 |
| 1 | 81 (10.8) | 104 (13.9) |  |  |
| 2 | 374 (49.9) | 346 (46.2) |  |  |
| 3 | 294 (39.3) | 299 (39.9) |  |  |
| CKD, n (%) | 125 (16.7) | 115 (15.4) | 0.526 | 0.036 |
| Congestive heart failure, n (%) | 258 (34.4) | 257 (34.3) | 1 | 0.003 |
| End stage renal disease, n (%) | 52 (6.9) | 49 (6.5) | 0.837 | 0.016 |
| Liver cirrhosis, n (%) | 28 (3.7) | 34 (4.5) | 0.517 | 0.04 |
| Cardiovascular diseases, n (%) | 514 (68.6) | 512 (68.4) | 0.956 | 0.006 |
| Hypertension, n (%) | 446 (59.5) | 426 (56.9) | 0.32 | 0.054 |
| Chronic lung disease, n (%) | 179 (23.9) | 180 (24) | 1 | 0.003 |
| Diabetes, n (%) | 66 (8.8) | 62 (8.3) | 0.782 | 0.019 |
| ARDS, n (%) | 1 (0.1) | 4 (0.5) | 0.37 | 0.069 |
| Coagulopathy, n (%) | 205 (27.4) | 215 (28.7) | 0.605 | 0.03 |
| Obesity, n (%) | 74 (9.9) | 69 (9.2) | 0.725 | 0.023 |
| Anemia, n (%) | 44 (5.9) | 41 (5.5) | 0.823 | 0.017 |
| Mean heartrate (median [IQR]) ^b^ | 89 [79,102] | 88 [79,101] | 0.913 | 0.008 |
| Mean MAP (median [IQR]) ^b^ | 71 [67,76] | 71 [66,76] | 0.618 | 0.009 |
| Platelet (median [IQR]) ^a^ | 186 [123,264] | 171 [118,265] | 0.1 | 0.03 |
| Creatinine (median [IQR]) ^a^ | 1.2 [0.8,2] | 1.2 [0.8,2.1] | 0.567 | 0.008 |
| Glucose (median [IQR]) ^a^ | 125 [103,155] | 122 [104,150] | 0.519 | 0.028 |
| Hemoglobin (median [IQR]) ^a^ | 10 [8.9,11.3] | 9.9 [9,11.2] | 0.925 | 0.026 |
| PT (median [IQR]) ^a^ | 14.7 [13.3,17] | 15 [13.7,17.6] | 0.011 | 0.001 |
| WBC (median [IQR]) ^a^ | 12 [8.3,17.5] | 11.9 [8.4,16.7] | 0.486 | 0.064 |
| Lactate (median [IQR]) ^a^ | 1.9 [1.3,2.8] | 1.7 [1.2,2.7] | 0.003 | 0.044 |
| PH (median [IQR]) ^a^ | 7.3 [7.3,7.4] | 7.4 [7.3,7.4] | 0.372 | 0.024 |
| Crystalloid input (median [IQR]) ^b^ | 2000 [500,4000] | 2250 [500,4000] | 0.821 | 0.005 |
| Urine output (median [IQR]) ^b^ | 520 [292,781] | 510 [290,737] | 0.555 | 0.001 |

**Abbreviations:** SOFA sequential organ failure assessment, SAPSII simplified acute physiology score II, GCS Glasgow coma score, MAP mean arterial pressure, AKI acute kidney injury, CKD chronic kidney disease, ARDS acute respiratory distress syndrome, RRT renal replacement therapy, PT prothrombin time, WBC white blood cell, SMD standardized mean difference.

^a^ The initial value during the first 24h after ICU admission.

^b^ The values were calculated during the first 24h after ICU admission.
