# Supplementary material for: Association between albumin infusion and outcomes in patients with acute kidney injury and septic shock
Source: Sci Rep. 2021 Dec 16;11:24083. doi: 10.1038/s41598-021-03122-0 (PMC8677767; doi:10.1038/s41598-021-03122-0)
Supplement: Supplementary file 4 — Supplementary Table S4. [file 41598_2021_3122_MOESM4_ESM.docx]

**Table S4. Dose-response relationship between albumin infusion and 28-day mortality**

| **Variables** | **HR (95%CI)** | **P** |
| --- | --- | --- |
| Albumin does (g/48h) |  |  |
| ≤25 | 0.83 (0.61-1.13) | 0.234 |
| 25-50 | 0.59 (0.39-0.88) | 0.009 |
| 50-100 | 0.85 (0.6-1.21) | 0.376 |
| 100-200 | 0.91 (0.63-1.3) | 0.592 |
| Age | 1.02 (1.01-1.03) | <0.001 |
| Weight | 0.99 (0.99-1) | 0.01 |
| White | 0.81 (0.64-1.01) | 0.064 |
| SOFA ^b^ | 1.07 (1.02-1.12) | 0.004 |
| GCS ^b^ | 1.02 (0.99-1.05) | 0.177 |
| SAPSII ^b^ | 1.01 (1-1.02) | 0.028 |
| RRT | 1.06 (0.73-1.55) | 0.742 |
| Ventilation | 0.77 (0.54-1.1) | 0.146 |
| AKI stage (per 1 stage increase) | 1.48 (1.21-1.82) | <0.001 |
| Cardiovascular diseases | 0.82 (0.65-1.03) | 0.093 |
| Hypertension | 0.81 (0.65-1.01) | 0.061 |
| Coagulopathy | 1.19 (0.94-1.5) | 0.15 |
| Obesity | 0.84 (0.53-1.34) | 0.476 |
| Anemia | 0.63 (0.38-1.04) | 0.07 |
| Mean heartrate ^b^ | 1.01 (1-1.01) | 0.021 |
| Mean MAP ^b^ | 0.96 (0.96-0.99) | 0.003 |
| Platelet ^a^ | 1 (1-1) | 0.012 |
| Bilirubin ^a^ | 1.03 (1.01-1.05) | 0.001 |
| Creatinine ^a^ | 1.02 (0.95-1.09) | 0.616 |
| Glucose ^a^ | 1 (1-1) | 0.022 |
| Hemoglobin ^a^ | 1.04 (0.98-1.11) | 0.156 |
| PT ^a^ | 1.01 (1-1.02) | 0.001 |
| WBC ^a^ | 1 (0.99-1.01) | 0.642 |
| Lactate ^a^ | 1.11 (1.08-1.15) | <0.001 |
| PH ^a^ | 1.91 (0.76-4.78) | 0.167 |
| Crystalloid does ^b^ | 1 (1-1) | 0.017 |
| Urine output ^b^ | 1 (1-1) | 0.614 |

**Abbreviations**: SOFA: sequential organ failure assessment, SAPSII: simplified acute physiology score II, GCS: Glasgow coma score, MAP: mean arterial pressure, AKI: acute kidney injury, PT prothrombin time, WBC white blood cell.

Multivariate cox proportional hazard models were used to assess dose-response relationship between albumin administration and 28-day mortality adjusting for confounders selected from a *P-*value < 0.05 in univariate analysis.

^a^ The initial values during the first 24h after ICU admission.

^b^ The values were calculated during the first 24h after ICU admission.
