# Supplementary material for: Association between albumin infusion and outcomes in patients with acute kidney injury and septic shock
Source: Sci Rep. 2021 Dec 16;11:24083. doi: 10.1038/s41598-021-03122-0 (PMC8677767; doi:10.1038/s41598-021-03122-0)
Supplement: Supplementary file 5 — Supplementary Table S5. [file 41598_2021_3122_MOESM5_ESM.docx]

**Table S5. Relationship between different types of colloids and 28-day mortality**

| **Variable** | **HR (95%CI)** | **P** |
| --- | --- | --- |
| Colloid type |  |  |
| Albumin use | 0.64 (0.54-0.76) | <0.001 |
| HES use | 0.56 (0.1-1.21) | 0.108 |
| Age | 1.01 (1.01-1.02) | <0.001 |
| Weight | 0.99 (0.99-1) | 0.006 |
| White | 0.84 (0.7-1.02) | 0.078 |
| SOFA ^b^ | 1.05 (1.01-1.09) | 0.015 |
| GCS ^b^ | 1 (0.98-1.02) | 0.823 |
| SAPSII ^b^ | 1.03 (1.02-1.03) | <0.001 |
| RRT | 0.93 (0.69-1.24) | 0.609 |
| Ventilation | 0.93 (0.69-1.25) | 0.631 |
| AKI | 0.69 (0.42-1.13) | 0.139 |
| AKI stage | 1.25 (1.06-1.47) | 0.007 |
| Cardiovascular diseases | 0.82 (0.67-0.99) | 0.044 |
| Hypertension | 0.87 (0.73-1.04) | 0.134 |
| Coagulopathy | 0.96 (0.79-1.17) | 0.695 |
| Obesity | 0.7 (0.46-1.07) | 0.097 |
| Anemia | 0.63 (0.4-0.99) | 0.043 |
| Mean heartrate ^b^ | 1.01 (1-1.01) | 0.001 |
| Mean MAP ^b^ | 0.96 (0.95-0.97) | <0.001 |
| Platelet ^a^ | 1 (1-1) | 0.495 |
| Bilirubin ^a^ | 1.03 (1.02-1.05) | <0.001 |
| Creatinine ^a^ | 1.02 (0.96-1.08) | 0.54 |
| Glucose ^a^ | 1 (1-1) | 0.406 |
| Hemoglobin ^a^ | 1.07 (1.03-1.12) | 0.001 |
| PT ^a^ | 1.01 (1-1.02) | <0.001 |
| WBC ^a^ | 1 (0.99-1.01) | 0.452 |
| Lactate ^a^ | 1.11 (1.08-1.15) | <0.001 |
| PH ^a^ | 0.63 (0.31-1.3) | 0.215 |
| Crystalloid input ^b^ | 1 (1-1) | 0.386 |
| Urine output ^b^ | 1 (1-1) | 0.658 |

**Abbreviations**: SOFA: sequential organ failure assessment, SAPSII: simplified acute physiology score II, GCS: Glasgow coma score, MAP: mean arterial pressure, AKI: acute kidney injury, PT prothrombin time, WBC white blood cell, HES hydroxyethyl starch.

Multivariate cox proportional hazard models were used to assess the relationship between different types of colloids and 28-day mortality adjusting for confounders with a *P-*value < 0.05 in univariate analysis.

^a^ The initial values during the first 24h after ICU admission.

^b^ The values were calculated during the first 24h after ICU admission.
